# Near-Surface Nanomechanics of Medical-Grade PEEK Measured by Atomic Force Microscopy

**DOI:** 10.3390/polym15030718

**Published:** 2023-01-31

**Authors:** Marco Bontempi, Rosario Capozza, Andrea Visani, Milena Fini, Gianluca Giavaresi, Alessandro Gambardella

**Affiliations:** 1 Scienze e Tecnologie Chirurgiche, IRCCS Istituto Ortopedico Rizzoli, Via di Barbiano 1/10, 40136 Bologna, Italy; 2 School of Engineering, Institute for Infrastructure and Environment, The University of Edinburgh, Thomas Bayes Road, Edinburgh EH9 3JL, UK; 3 Scientific Direction, IRCCS Istituto Ortopedico Rizzoli, Via di Barbiano 1/10, 40136 Bologna, Italy

**Keywords:** PEEK, nanoindentation, biopolymers, orthopedics, nanomechanical mapping, atomic force microscopy, elasticity

## Abstract

Detecting subtle changes of surface stiffness at spatial scales and forces relevant to biological processes is crucial for the characterization of biopolymer systems in view of chemical and/or physical surface modification aimed at improving bioactivity and/or mechanical strength. Here, a standard atomic force microscopy setup is operated in nanoindentation mode to quantitatively mapping the near-surface elasticity of semicrystalline polyether ether ketone (PEEK) at room temperature. Remarkably, two localized distributions of moduli at about 0.6 and 0.9 GPa are observed below the plastic threshold of the polymer, at indentation loads in the range of 120–450 nN. This finding is ascribed to the localization of the amorphous and crystalline phases on the free surface of the polymer, detected at an unprecedented level of detail. Our study provides insights to quantitatively characterize complex biopolymer systems on the nanoscale and to guide the optimal design of micro- and nanostructures for advanced biomedical applications.

## 1. Introduction

Polyether ether ketone (PEEK) is a semicrystalline polymer with excellent biocompatibility, chemical resistance, a high melting temperature (340 °C), radiation and sterilization resistance and radiolucency [1,2]. Its elastic modulus is 3.5–4.0 GPa, making it a good candidate for a variety of biomedical purposes, such as a biomaterial for spinal cages, cranial reconstruction and dental implant applications [3,4,5,6,7,8]. In spite of this, its nonexact match with the elastic moduli of cortical bone (several GPa) and the lack of surface bioactivity and osseointegration hinder its orthopedic applications [2,9,10]. In recent years, advances in nanotechnology have promoted novel strategies to increase the bioactivity of PEEK through modification of surface composition and/or morphology, aiming at obtaining implants with antibacterial properties, osteoconduction and improved mechanical strength [11,12,13,14,15,16,17,18,19,20]. In particular, modifications of surface micro- and nanotexture can control cell differentiation via affecting cell-generated physical forces, thus improving substrate osseointegration [21]. Methods to modify the PEEK surface mainly include plasma-immersion processes to reach the desired topological structures, with positive effects on cell–substrate interactions [22,23].

Modification of physical properties (e.g., surface morphology and stiffness) is as important as chemical composition for the design of an implant biomaterial [10]. A growing body of evidence has proven that substrate stiffness plays a significant role in regulation of cell behavior including differentiation, proliferation, migration and apoptosis [10,24,25].

Thus, an ideal characterization tool should be able to discriminate stiffness gradients along distances comparable to or smaller than cellular dimensions. Such knowledge is fundamental to explore the efficiencies of various functional implant surfaces and develop suitable structure–property relationships in consideration of submicron debris, wear and cell adhesion [26,27,28,29,30,31,32].

However, studies of the nanomechanical behavior of PEEK are lacking due to the inherent difficulty of investigating polymers at this length scale. Despite many bulky nanoindentation studies, the near-surface mechanics of the polymer on the micro- and nanoscale are still lacking. Advanced characterization of the mechanics of complex biopolymers typically involves atomic force microscopy-based nanoindentation [33,34]. This technique uses a tip–cantilever system to probe the mechanics of the surface at selected points within a given topographic region of the sample. The nanometrically sharp AFM tip is brought in contact with the sample surface, and the deflection of the cantilever is measured as a function of the distance (loading–unloading-indentation curve). The indentation modulus (or stiffness) *E* of a sample can then be extracted from the curve by means of appropriate fitting models. In addition, a grid pattern of curves can be acquired within a given topography of the sample, providing a stiffness map of the same area. Due to the extreme spatial resolution and force sensitivity of AFM, stiffness maps are potentially capable of capturing local gradients in the elastic properties of samples [35,36,37].

AFM-based nanoindentation of complex biopolymers focused on loading-unloading indentation measurements under different loading conditions to study the nanomechanical properties and viscoelasticity of samples [33,34,37,38]. In this context, the potential of this technique has been explored to characterize the plasticity of PEEK up to nanometric resolution [38,39,40]. Hence, there is a lack of studies exploring the nanomechanics of PEEK under elastic indentation regime, where small (<1 µN) loading forces and indentation depths much smaller than 100 nm are involved [33]. Typically, for small loading forces immediately after contact (i.e., along the loading curve), the tip–polymer interaction is poorly affected by adhesion, plastic deformations and time-dependent phenomena; only elastic deformations will be present and elastic theories, e.g., Hertz theory, can be used [41]. Furthermore, performing nanoindentations at high loading rates allows minimization of the residual imprints of nanoindentation; as a consequence, loading–unloading will be dominated by elastic behavior with negligible irreversible deformation [32]. Therefore, considering suitably small forces and reversible surface deformation simplifies the experiments and, at the same time, enables the study of PEEK under conditions relevant to biological systems.

This work explores the potential of AFM in investigating the elasticity of PEEK at the nanoscale. To this aim, a standard experimental setup with nanometrically sharp tips (spherical shape with curvature radius of *R* = 10 nm) and moderate cantilever stiffness (from about 9 to 29 N/m) was used. Nanoindentations were operated on a grid pattern within 5 × 5 µm^2^ large topographies of the sample, with loading–unloading curves extracted at each point of the grid. Besides *E*, other useful quantities derived from the curves could be mapped, such as the maximum indentation depth *h_max_* and the elasticity index, 0 < *η_el_* < 1; the latter is defined as the ratio between the areas under the unloading and loading curves and indicates the degree of elasticity of the deformation at each point of indentation [35,42]. We show that these three quantities, which can be obtained simultaneously from a single indentation grid, provide comprehensive characterization of the nanomechanics of PEEK and promote AFM-based nanomechanical mapping as a mean to identify and quantify the spatial localization of the moduli originated by the contributions of different surface phases of the polymer, as well as to monitor changes of such distributions due to chemical and physical modification of the surface.

## 2. Materials and Methods

### 2.1. Samples, AFM Setup and Calibration

The PEEK samples were medical-grade sheets (10 × 10 × 6 mm height; Direct Plastics Ltd., Sheffield, UK) with semimachined surfaces, as described previously [43]. AFM measurements were performed by a NT-MDT (Moscow, Russia) system equipped with an upright optical microscope. NSG10 and NSG30 tips (NT-MDT, Moscow, Russia) with resonant frequencies in the range of 140–390 kHz were used. Topographies were taken at a 256 × 256-pixel resolution and acquired in tapping mode of operation. The cantilever stiffness *k* was measured according to Sader [35], a procedure implemented into the acquisition software (NOVA, MT-MDT, Moscow, Russia). The cantilever deflection sensitivity was calibrated from a set of indentation curves previously obtained under hard-contact regime on a clean and nanometrically flat silica slice (~80 GPa in stiffness); such procedure prevents from damages the tips actually used. PEEK slices were mounted on the sample stage and characterized both topographically and by extraction of force curves. Before and after mapping, the integrity of the tips was checked via z-axis calibration on a TGS1 calibration grating (NT-MDT, Moscow, Russia; grid TGZ1 with a height of (21 ± 1) nm). Two-dimensional arrays of *F-d* curves (1000 points each) were acquired at six non-overlapped 5 × 5 µm^2^ areas on 20 × 20 grids. Note that the spatial resolution of the mapping is equal to the distance between the indents. In this work, a distance of 5000 nm/20 = 250 nm was set according to theoretical considerations [44,45] and to reduce the acquisition time of a single map (<1 h). The loading/unloading rate was fixed at 500 nms^−1^ for all indentation tests. Several survey curves were taken before mapping to verify that *h_max_* fell in the range suitable to the model used (see later).

### 2.2. Indentation Modulus Calculation

**Figure 1:**
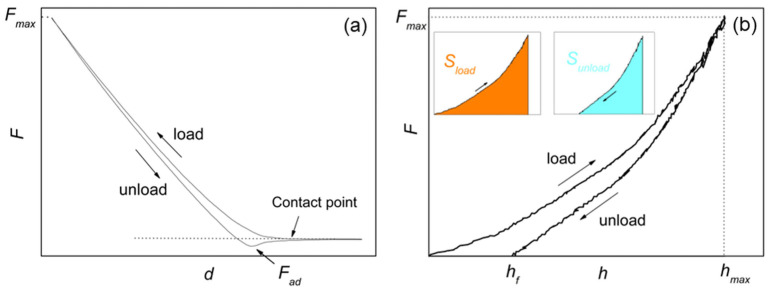
(**a**) *F-d* curve with indication of the contact point and adhesion force *F_ad_*. (**b**) Force-penetration (*F-h*) curve equivalent to *F-d*, with indication of the effective and maximum penetration depths *h_f_* and *h_max_* and the maximum force load *F_max_* = *F*(*h_max_*). In the inset, the areas under the load (*S_load_*) and unload (*S_unload_*) curves are evidenced in colors.

Figure 1a illustrates a typical loading–unloading or force–distance (*F-d*) curve, with indication of the adhesion force *F_ad_* (typically, <10 nN in our measurements) and contact point. The calibration operation converts the *F-d* into the corresponding *F-h* curve (Figure 1b), where *h* is the penetration depth. Referring to the areas in colors in Figure 1b, *η_el_* = *S_unload_*/*S_load_* for each curve was calculated; for a totally plastic sample, *η_el_* = 0, while for a totally elastic sample, *η_el_* = 1 [35].

According to the Hertz theory, in case of a spherical indenter of radius *R,* purely elastic deformation and negligible adhesion force *F_ad_*, the Young’s modulus *E* of an isotropic, homogeneous sample will follow the well-known *h*^−3/2^ dependence [35,44]. Following a previous work of this group [41], in this study *E* is calculated by the modified Hertz formula introduced by Kontomaris [44,45], which accounts for indentations comparable to or higher than the dimension of the indenter (*h* ≥ *R*). This modified Hertz formula differs from the original by a factor depending on *h/R*; if *h* < *R*, the original Hertz formula is obtained. If *h* >> *R*, linear dependence is obtained [45].

### 2.3. Data Processing and Analysis

Indentation maps obtained by AFM were processed using an assembly of different algorithms implemented as a Python module [46] that could be adapted to a user’s computational needs, as described previously [43]. In brief, the software processes the *F-d* curves, aligning them and separating loading and unloading curves to generate *F-h* curves. Then, the indentation modulus *E* was calculated applying the modified Hertz formula of Section 2.2 to the loading part of the *F-h* curve. The Poisson’s ratio of PEEK (*ν* = 0.38) and the radius *R* enters into calculation of *E*. Following previous studies [33,43], an uncertainty of 10% on these quantities was propagated. Then, the uncertainty Δ*E* and the correlation coefficient of the fitting *r*^2^ were computed. Afterward, to assess the correctness and the consistency of the data, rejection criteria based on *r*^2^ and Δ*E/E* can be tailored according to the given experimental context. In the present study, the conditions *r*^2^ > 0.95 and Δ*E/E* < 30% were required; this caused -in average- rejection of <3% of the acquired curves.

## 3. Results

### 3.1. Checking the Elastic Response of the Surface

**Figure 2:**
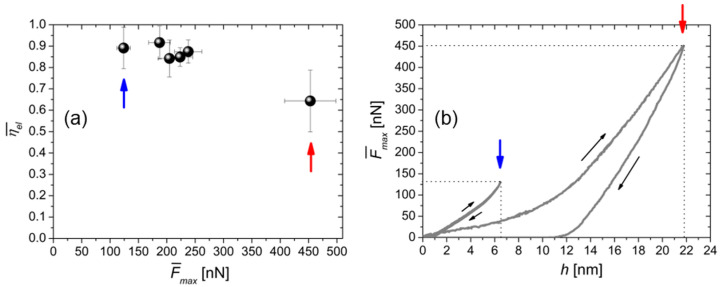
(**a**) Plot of ${\bar{\eta }}_{el}$ vs. ${\bar{F}}_{max}$. (**b**) Two representative *F-h* curves taken from the maps recorded respectively at ${\bar{F}}_{max}$ = (120 ± 11) nN and (453 ± 49) nN and corresponding to ${\bar{\eta }}_{el}$ = (0.891 ± 0.096) and (0.643 ± 0.146), namely the first and last points of the graph in (**a**). Blue and red arrows are a guide to the eye.

PEEK indentations by cantilevers with different stiffness produced, in general, different mechanical responses. Thus, cantilevers with different *k* were used while *η_el_* was continuously monitored during data acquisition to ensure that the condition *η_el_* ~ 1 (elastic behavior) was fulfilled. Furthermore, an uncertainty of 10–15% on ${\bar{F}}_{max}$ was determined by statistical analysis; such statistical fluctuations are due to instrumental and local contact instabilities. For each map, ${\bar{\eta }}_{el}$ and the corresponding ${\bar{F}}_{max}$ were extracted as an average. Assuming homogeneous mechanical response of the surface, the six ${\bar{\eta }}_{el}$(${\bar{F}}_{max})$ values were plotted in Figure 2a at increasing ${\bar{F}}_{max}$. Note that elastic deformations were produced up to a load of ~450 nN, which represents the transition point for plastic deformation in this work (*η_el_*~0.6). For the sake of clarity, two representative *F-h* curves corresponding to the lowest and highest loads (the first and last points of Figure 2a, respectively), are shown (Figure 2b). Note that the *h_max_* values (6.3 nm and 21.8 nm respectively) fall in the range of validity of the modified Hertz model used to fit the data (Section 2.2). On the other hand, the effective penetration depth *h_f_* is less than 1 nm for the left curve, while exceeds 12 nm for the right one. The larger *h_f_* value implies a lower fitting quality with higher deformations, as only elastic deformations were assumed, as discussed in the following.

### 3.2. Force-Mapping Results

**Figure 3:**
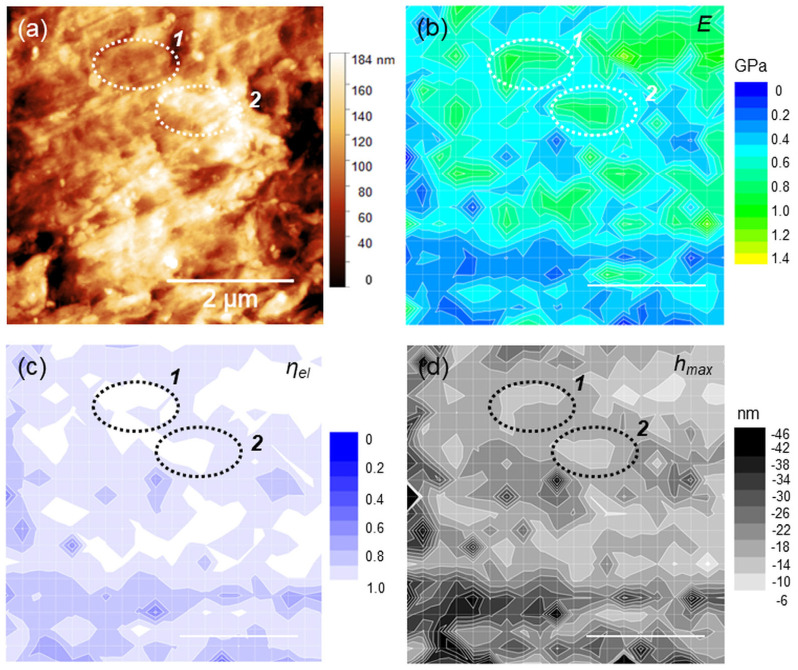
(**a**) 5 × 5 µm^2^ topography with indication of two features of interest (1 and 2); (**b**–**d**) *E*, *η_el_* and *h_max_* maps of the same region.

**Figure 4:**
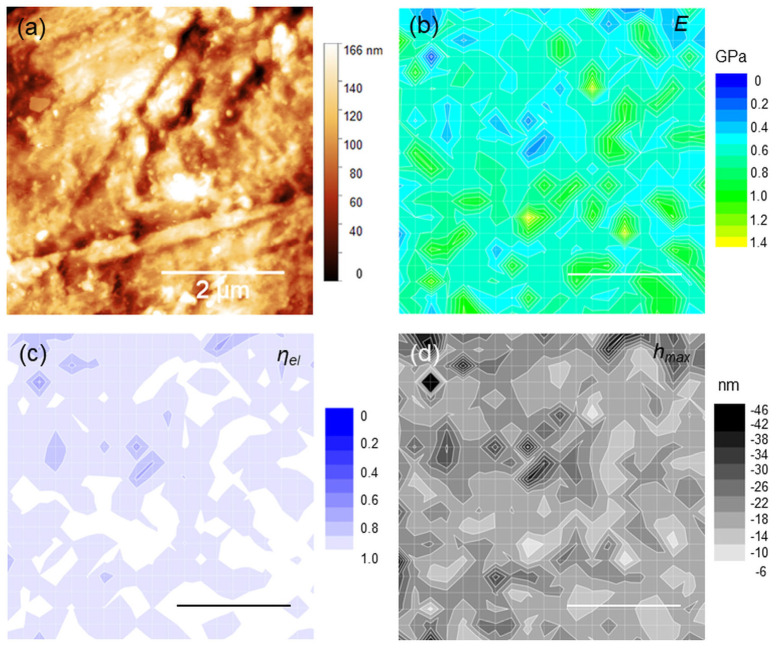
(**a**) 5 × 5 µm^2^ topography of a different sample region; (**b**–**d**) corresponding *E*, *η_el_* and *h_max_* maps.

Sample areas 5 × 5 µm^2^ large were considered for the AFM analysis, based on a preliminary survey of the surface and previous studies [34,40]. In Figure 3a, a topographic image (roughness 15.6 nm, peak-to-peak height 184 nm) is shown along with the corresponding maps of *E*, *η_el_* and *h_max_*. (Figure 3b–d). By way of example, two features *1* and *2* are evidenced in the topography. These features are separated by a step height of (20 ± 5) nm along the z-axis. At the same positions, two distinct regions or “islands” of localized moduli at ~0.9 GPa can be observed (Figure 3b). Significantly, both the regions exhibited purely elastic response (*η_el_*~0.95), with the same deformation *h_max_*~12 nm (Figure 3c,d). Although similar correspondences between topographies and maps can be observed likewise at different positions, they are—in general- quite elusive. This is the case of Figure 4 (roughness 17.5 nm, peak-to-peak height 166 nm), where several small (<1 µm) islands were detected; remarkably, these islands appear prevalently elastic in nature and correspond to the smallest *h_max_* within the image. Nevertheless, the topographic step heights measured in correspondence of the islands are too small (<5–10 nm) to be significant in relation to the image roughness.

**Figure 5:**
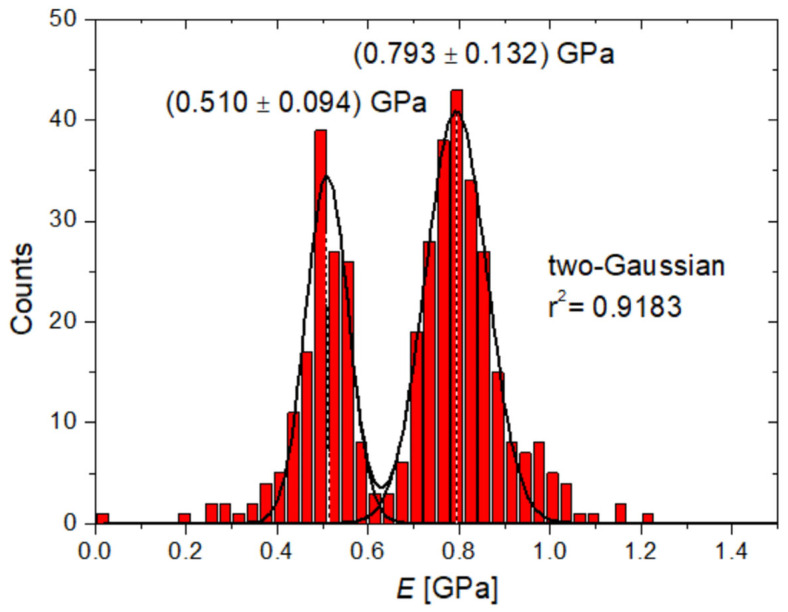
Representative statistical histogram of the moduli extracted from the *E* map measured at ${\bar{F}}_{max}$ = (187 ± 19) nN, with indication of the corresponding two-Gaussian fitting.

For quantitative analysis, statistical histogram plots of moduli were extracted from each map, as in Figure 5. To account for the presence of two distinct peaks in the distributions, two-Gaussian functions were used to fit the data, corresponding to lower and higher distributions of moduli with calculated peaks at *E*_1_ and *E*_2_, respectively. The results obtained for the six regions considered are summarized in Table 1 at increasing loads.

It should be noted that a rigorous mutual comparison between the reported moduli is not possible, as they were acquired at different ${\bar{F}}_{max}$ values. Nevertheless, the fair agreement between data and fitting functions (*r*^2^ ~ 0.9) allows us to infer some quantitative conclusions. The statistical distribution of moduli extracted from the map corresponding to the lowest applied load (120 nN) was fitted by a Gaussian, and the corresponding modulus (1.37 ± 0.85) GPa was provided for completeness. However, a bimodal distribution of moduli was clearly observed for the maps acquired at higher loads. This corroborates the presence of a spatial localization of the measured moduli as evidenced in Figure 3 and Figure 4. Both *E*_1_ and *E*_2_ tend to increase as ${\bar{F}}_{max}$ increases. The increase of *E*_1_ was slower than the *E*_2_ (2.3 × 10^−3^ against 3.7 × 10^−3^ GPa/nN). We hypothesized such increase is due to viscoelastic effects, as well as to the progressive inability of the elastic model to describe the indentation at large deformations. This is suggested by the fact that the *E*_1_ distribution is broader than the *E*_2_ distribution (28% compared to 14%), implying that the localization in islands mainly concerned the high moduli. Despite displaying the same trends described above, the point at 453 nN was not considered as the low *r*^2^ suggested that the inelastic threshold is about to be reached and, as a consequence, the modified Hertz model is not suitable for data analysis.

## 4. Discussion

### 4.1. Some Methodological Remarks: Choise of the Cantilever, Measured Stiffness

This study aimed at characterizing the nanoscale mechanical properties of medical-grade PEEK by AFM-based nanoindentation; the quantities relevant to the analysis (*h_max_*, *η_el_* and *E*) were obtained simultaneously from each indentation point on the surface assuming only elastic deformation. Some points can be discussed further on the basis of the experimental results.

Generally speaking, models of elastic deformation require some assumptions to be made such as isotropic, homogeneous samples, negligible *F_ad_* and infinitesimal indentations, i.e., indentation depths much smaller than the dimension of the indenter [35]. However, increasing the dimension of the indenter beyond several nanometers implies loss of resolution in topographic mode while, at the same time, cantilevers with high (>100 N/m) stiffness and consequently high loading forces (typically, 50 µN [39]) should be used to reduce noise-to-signal ratio and obtain compliant results. As a result, indentation becomes an irreversible process, or, in other words, residual indent is found on the sample, and elastic–plastic deformation regime should be assumed to fit the data [38]. In our work, the usage of nanometrically sharp tips for better spatial resolution implies *h*~*R*, therefore the modified Hertz model of Section 2.2 can be used to fit the data [41,42,43]. This allowed to numerically process nanometric indentations into PEEK, while the usage of low-stiffness cantilevers allows investigating the polymer mechanical response to small applied forces. Finally, an observation should be made as concerns our findings for smaller moduli compared to traditional bulky measurements. In this study, which focused on penetration depths comparable to the size of the indenter, only the very few layers below the free surface of the polymer were probed. Thus, it is possible that small nanoindentations would measure smaller moduli when compared to deeper nanoindentations, as the latter specifically probe a large enough volume of bulk to render the contribution from a mobile surface layer and energy dissipation insignificant [38].

### 4.2. Considerations on the Nanoscale Mechanics of PEEK

The basic structure of semicrystalline PEEK in the crystalline and amorphous phases has been recently studied as a function of temperature by contact-resonance AFM technique [38]. This technique exploits the mechanical vibration response of an AFM cantilever to probe mechanical properties by sensing the resonance frequency of the cantilever which is oscillating on the test sample. As a result, the local viscoelastic properties of materials at the nanoscale are probed. The main results of this study were the differentiation between the amorphous and crystalline phases, and the localization of the storage moduli as two distinct values; these findings were clearer at temperatures in the range of 80–180 °C, where the amorphous phase markedly separated from the crystalline phase because of the softening of the polymer with increasing temperature. Remarkably, at 30 °C, the resonance peaks were too narrow to be resolved as bimodal distribution of moduli.

In this respect, one may suggest that the measure of the modulus based on the analysis of the force curve has enough resolution to distinguish the crystalline and amorphous phases at room temperature. This fact underlines the sensitivity of nanomechanical force spectroscopy compared to other force–volume techniques routinely used for characterizing complex biopolymers [37,38]. The description of the bare PEEK surface provided in this work, therefore, represents a first validation step for elucidating changes in the spatial distribution of stiffness that may occur in response to modifications in composition and/or texture.

The morphological model of PEEK comprises stacks of crystalline lamellae separated by interlamellar amorphous layers, with adjacent lamellar stacks separated by regions of interfibrillar amorphous material [47]. Such alternance at the surface of crystalline lamellae and amorphous regions suggests an explanation for the different localizations of the “high” and “low” moduli measured in this work. Moreover, the elastic-like character of the crystalline portion of the surface has also been highlighted recently in studies on the correlations between structure and mechanical properties of polyethylene by AFM nanoindentation experiments and molecular dynamics simulations [48]. Those authors found that the shape of each force curve was strongly position-dependent, although the intrinsic inhomogeneity of crystalline structures made it difficult to discriminate between different local structures and how they affect the nanomechanical properties of a complex biopolymer. Thus, at this stage, we have no evidence of correlation between the spatial distribution of step heights observed in correspondence of the localized moduli and the various interpretations of structural data [47,49]. Recently, a comparative analysis has proven that Raman spectroscopy is superior to X-ray diffraction and other techniques in determination of the local crystallinity on the polymer surface [49]. Thus, further investigation should involve Raman spectroscopy to resolve the crystalline structure at the PEEK surface and to correlate it with the AFM findings.

## 5. Conclusions

This study characterized the nanomechanical properties of PEEK in the elastic regime using a simple atomic force microscopy experimental setup. Maps of stiffness, penetration depth and elasticity index acquired simultaneously on a given sample topography provided information about the localization of crystalline and amorphous structures in response to a few hundreds nN loads. These findings provide a route to improve our knowledge of the interrelationships between mechanics and micro/nanomorphological and/or compositional characteristics, which would be useful for study of the interface between the polymer surface and the biological environment. The methodology detailed here is potentially applicable to the studies of various complex biopolymer surfaces.

## Figures and Tables

**Table 1:** Means and standard deviations calculated from the two-Gaussian fittings of the distributions of moduli extracted from the maps.

${\bar{F}}_{max}$ ± *σ* [nN]	*E*_1_ ± *σ*_1_ [GPa]	*E*_2_ ± *σ*_2_ [GPa]	*r* ^2^
120 ± 11	-	(1.37 ± 0.85) ^1^	(0.8371) ^1^
187 ± 19	0.510 ± 0.094	0.793 ± 0.132	0.9183
205 ± 21	0.509 ± 0.210	0.828 ± 0.106	0.8859
223 ± 22	0.634 ± 0.180	0.917 ± 0.142	0.9306
238 ± 25	0.626 ± 0.160	0.982 ± 0.107	0.9031
453 ± 49	1.04 ± 0.374	1.98 ± 1.37	0.6944

^1^ This value corresponds to one-Gaussian fitting.

## Data Availability

Not applicable.
